# Supplementary material for: Gut Microbiota Profile of Obese Diabetic Women Submitted to Roux-en-Y Gastric Bypass and Its Association with Food Intake and Postoperative Diabetes Remission
Source: Nutrients. 2020 Jan 21;12(2):278. doi: 10.3390/nu12020278 (PMC7071117; doi:10.3390/nu12020278)
Supplement: Supplementary file 1 [file nutrients-12-00278-s001.pdf]

Supplementary files:

Table S1. Clinical, anthropometric, body composition, biochemical and daily food intake features from obese patients with postoperative type 2 diabetes remission, before and after 3 and 12 months of Roux en-Y gastric bypass

Variable	Preoperative	3-month Postoperative	12-month Postoperative	*p* value*	*p* value#	*p* value@
BMI (kg/m²)	48.00 ± 5.04	39.50 ± 3.26	33.10 ± 3.63	**<0.050**	**<0.050**	**<0.050**
Body weight (kg)	113.00 ± 18.20	93.40 ± 13.10	78.00 ± 10.30	**<0.050**	**<0.050**	**<0.050**
FM by DXA (%)	52.6 ± 9.55	48.70 ± 5.16	41.40 ± 6.01	0.180	**0.055**	**<0.050**
WC (cm)	126.00 ± 13.90	111.00 ± 10.70	101.00 ± 9.56	**<0.050**	**<0.050**	**<0.050**
HC (cm)	142.00 ± 9.81	126.00 ± 9.05	111.00 ± 7.89	**<0.050**	**<0.050**	**<0.050**
WC:HC ratio (cm)	0.89 ± 0.08	0.88 ± 0.06	0.91 ± 0.04	0.310	0.450	0.250
SBP (mmHg)	163.00 ± 24.00	148.00 ± 19.50	139.00 ± 22.00	**0.055**	**<0.050**	0.360
DBP (mmHg)	104.00 ± 24.10	92.40 ± 12.50	91.50 ± 16.10	0.250	**<0.050**	0.800
HDL (mg/dL)	45.00 ± 12.70	43.40 ± 10.40	52.20 ± 14.10	0.440	**<0.050**	0.058
LDL (mg/dL)	103.00 ± 31.90	86.00 ± 24.80	84.20 ± 18.20	0.310	0.230	0.840
VLDL (mg/dL)	24.2- ± 6.09	19.00 ± 5.07	17.20 ± 3.54	0.180	**<0.050**	0.170
TC (mg/dL)	172.00 ± 38.50	148.00 ± 30.00	154.00 ± 17.70	0.310	0.270	0.670
Triglyc. (mg/dL)	121.00 ± 30.10	95.40 ± 24.90	85.60 ± 17.40	0.250	**<0.050**	0.110
Hyperchol. (%)	50.00	12	0	0.250	0.130	**1.000**
Hypertrigl. (%)	62.00	12	0	0.130	0.074	**1.000**
MetS (%)	100.00	50	25	0.130	<0.050	0.620
C peptide (ng/mL)	4.36 ± 0.71	2.53 ± 0.44	2.29 ± 0.50	**<0.050**	**<0.050**	0.150
FG (mg/dL)	198.00 ± 74.30	88.80 ± 9.1	83.20 ± 9.18	**<0.050**	**<0.050**	**<0.050**
A1c (%)	8.90 ± 2.14	5.87 ± 0.50	5.40 ± 0.39	**<0.050**	**<0.050**	**<0.050**
FI (mUI/L)	18.60 ± 6.43	8.30 ± 4.54	6.0 ± 2.21	**<0.050**	**<0.050**	0.470
HOMA-IR (%)	8.57 ± 4.41	1.81 ± 1.05	1.36 ± 0.57	**<0.050**	**<0.050**	0.300
Metformin use (%)	88.00	12	0	<0.050	<0.050	**1.000**
Energy (kcal/d)	1590.00 ± 264.00	1090.00 ± 236.00	1330.00 ± 407.00	**<0.050**	0.150	0.250
Protein (g/d)	70.40 ± 17.90	53.10 ± 9.84	62.70 ± 16.60	0.055	0.380	0.200
Carbohydrate (g/d)	201.00 ± 22.10	124.00 ± 36.60	164.00 ± 49.30	**<0.050**	**0.055**	0.150
Fat (g/d)	60.00 ± 14.60	44.20 ± 11.00	37.80 ± 9.23	**<0.050**	**<0.050**	0.310
Saturated fat (g/d)	15.80 ± 4.27	11.90 ± 4.26	11.40 ± 2.09	0.150	**<0.050**	1.000
MUF (g/d)	16.90 ± 4.21	11.60 ± 3.72	9.78 ± 3.29	0.055	**<0.050**	0.460
PUF (g/d)	12.20 ± 2.72	8.52 ± 2.61	5.91 ± 1.19	**<0.050**	**<0.050**	**<0.050**
Fiber (g/d)	15.00 ± 4.14	10.50 ± 4.00	11.60 ± 2.94	0.055	0.310	0.640
IF (g/d)	3.26 ± 0.10	2.60 ± 1.24	3.00 ± 0.99	0.250	0.800	0.640
SF (g/d)	1.77 ± 0.55	1.36 ± 0.81	1.92 ± 0.80	0.200	0.950	0.250
F:L ratio (g/d)	0.27 ± 0.13	0.24 ± 0.09	0.31 ± 0.08	0.550	0.460	0.150
B12 (mcg/d)	0.86 ± 0.21	0.73 ± 0.23	0.86 ± 0.52	0.180	0.300	0.940

Legend. Data assessed in eight patients are expressed in mean ± standard deviation or absolute percentage and significant differences between periods are highlighted in bold, according to the

follows comparisons: *3-month postoperative (n = 20) vs. preoperative (n = 25); #12-month postoperative (n = 14) vs. preoperative; @3-month postoperative vs. 12-month postoperative. Most variables were significantly lower 3 and/or 12 months after surgery than at preoperative, except the waist:hip ratio, low-density lipoprotein cholesterol, total cholesterol, metabolic syndrome (frequency), protein (borderline), fiber (borderline), insoluble fiber, soluble fiber, fiber:lipid ratio, and vitamin B12 intake. Missing data: one patient for fasting C peptide. A1C, glycated hemoglobin; B12, vitamin B12; BMI, body mass index; DBP, Diastolic blood pressure; DXA, dual-energy X-ray absorptiometry; F:L ratio, fiber to lipid ratio; FG, fasting glucose; FI, fasting insulin; FM, fat mass; HC, hip circumference; HDL, high-density lipoprotein cholesterol; Hiperchol., hypercholesterolemia; Hypertrigl., hypertriglyceridemia; HOMA-IR, insulin resistance index; IF, insoluble fiber; LDL, low-density lipoprotein cholesterol; MetS, metabolic syndrome; MSF, monounsaturated fat; PUF, polyunsaturated fat; SBP, Systolic blood pressure; SF, soluble fiber; TC, total cholesterol; Triglyc., triglycerides; VLDL, very-low-density lipoprotein cholesterol; WC, waist circumference.

Table S2. Clinical, anthropometric, body composition, biochemical, and daily food intake features from obese patients without postoperative type 2 diabetes remission, before and after 3 and 12 months of Roux en-Y gastric bypass

**Clinical variable**	**Preoperative**	**3-month Postoperative**	**12-month Postoperative**	***p* value***	***p* value#**	***p* value@**
BMI (kg/m^2)	43.30 ± 4.61	35.70 ± 4.04	32.10± 3.67	**<0.050**	**<0.050**	**<0.050**
Body weight (kg)	115.00 ± 14.30	95.00 ± 13.20	85.50 ± 11.60	**<0.050**	**<0.050**	**<0.050**
FM by DXA (%)	50.80 ± 4.72	44.70 ± 7.96	41.60 ± 8.17	**<0.050**	**<0.050**	0.160
WC (cm)	124.00 ± 17.10	111.00 ± 17.30	105.00 ± 14.40	**<0.050**	**<0.050**	0.160
HC (cm)	135.00 ± 8.80	119.00 ± 11.10	114.00 ± 8.42	**<0.050**	**<0.050**	0.220
WC:HC ratio (cm)	0.92 ± 0.09	0.93 ± 0.08	0.91 ± 0.06	0.830	1.000	0.310
SBP (mmHg)	153.00 ± 31.30	138.00 ± 12.00	136.00 ± 20.90	0.310	0.160	1.000
DBP (mmHg)	98.20 ± 25.90	85.00 ± 13.50	90.00 ± 14.70	0.250	0.220	0.310
HDL (mg/dL)	44.80 ± 10.60	44.70 ± 10.90	55.00 ± 12.20	0.920	0.160	**<0.050**
LDL (mg/dL)	126.00 ± 26.90	95.70 ± 30.50	86.00 ± 21.10	0.140	0.094	0.440
VLDL (mg/dL)	28.20 ± 10.30	21.50 ± 8.41	16.30 ± 2.16	0.200	0.059	0.140
TC (mg/dL)	199.00 ± 22.20	162.00 ± 35.60	157.00 ± 24.40	0.094	0.063	1.000
Triglyc. (mg/dL)	140.00 ± 52.40	108.00 ± 42.00	81.50 ± 10.80	0.250	0.063	0.180
Hyperchol. (%)	50.00	33.00	17.00	1.000	0.480	1.000
Hypertrigl. (%)	83.00	17.00	17.00	0.130	0.130	1.000
MetS (%)	100.00	83.00	50.00	1.000	0.250	0.480
C peptide (ng/mL)	3.47 ± 1.57	2.68 ± 1.05	2.38 ± 0.58	0.380	0.190	0.250
FG (mg/dL)	268.00 ± 70.30	120.00 ± 32.50	106.00 ± 14.00	**<0.050**	**<0.050**	0.310
A1c (%)	9.25 ± 1.33	6.43 ±0.543	6.20 ± 0.32	**<0.050**	**<0.050**	0.590
FI (mUI/L)	12.20 ± 2.78	7.05 ±1.46	5.70 ± 1.60	0.120	**<0.050**	0.120
HOMA-IR (%)	7.89 ± 2.24	1.77 ± 0.44	1.46 ± 0.34	0.120	**<0.050**	0.250
Metformin use (%)	100.00	17.00	33.00	0.074	0.130	1.000
Energy (kcal/d)	1550.00 ± 326.00	890.00 ± 253.00	1210.00 ± 296.00	**<0.050**	**<0.050**	0.063
Protein (g/d)	62.80 ± 9.83	43.20 ± 21.00	59.10 ± 19.80	0.063	1.000	0.063
Carbohydrate (g/d)	201.00 ± 44.40	106.00 ± 24.00	148.00 ± 48.60	**<0.050**	**<0.050**	**<0.050**
Fat (g/d)	56.80 ± 16.20	33.20 ± 11.20	42.50 ± 7.20	**<0.050**	0.063	0.160

Saturated fat (g/d)	15.00 ± 4.14	8.91 ± 3.87	11.80 ± 0.85	0.063	0.220	0.094
MUF (g/d)	15.80 ± 5.25	9.47 ± 3.62	10.60 ± 2.74	**<0.050**	**<0.050**	0.440
PUF (g/d)	11.40 ± 3.86	8.14 ± 2.01	6.63 ± 0.90	**<0.050**	0.094	0.220
Fiber (g/d)	16.50 ± 8.31	7.9 ± 2.15	9.45 ± 2.98	**<0.050**	0.063	0.160
IF (g/d)	3.70 ± 1.46	2.19 ± 0.80	2.59 ± 1.25	**<0.050**	**<0.050**	0.440
SF (g/d)	3.20 ± 2.09	1.18 ± 0.49	1.38 ± 0.73	**<0.050**	**<0.050**	0.560
F:L ratio (g/d)	0.30 ± 0.14	0.25 ± 0.05	0.22 ± 0.06	0.840	0.220	0.690
B12 (mcg/d)	0.90 ± 0.23	0.88 ± 0.44	0.76 ± 0.42	1.000	0.310	0.310

Legend. Data assessed in six patients are expressed in mean ± standard deviation or absolute percentage and significant differences between periods are highlighted in bold, according to the follows comparisons: *3-month postoperative (n = 20) vs. preoperative (n = 25); #12-month postoperative (n = 14) vs. preoperative; @3-month postoperative vs. 12-month postoperative. All the body composition variables improved at the postoperative, except the hip to waist ratio; but most the clinical and biochemical variables did not change, except by a decrease in fasting glucose, glycated hemoglobin, fasting insulin, and insulin resistance after 3 and/or 12 months of surgery and an increase of the high-density lipoprotein cholesterol when comparing these postoperative time points. Food intake decreased after 3 and/or 12 months of surgery, except protein, saturated fat, fiber to lipid ratio, and vitamin B12. Missing data: one patient for fasting C peptide. A1C, glycated hemoglobin; B12, vitamin B12; BMI, body mass index; DBP, Diastolic blood pressure; DXA, dual-energy X-ray absorptiometry; F:L ratio, fiber to lipid ratio; FG, fasting glucose; FI, fasting insulin; FM, fat mass; HC, hip circumference; HDL, high-density lipoprotein cholesterol; Hiperchol., hypercholesterolemia; Hypertrigl., hypertriglyceridemia; HOMA-IR, insulin resistance index; IF, insoluble fiber; LDL, low-density lipoprotein cholesterol; MetS, metabolic syndrome; MSF, monounsaturated fat; PUF, polyunsaturated fat; SBP, Systolic blood pressure; SF, soluble fiber; TC, total cholesterol; Triglyc., triglycerides; VLDL, very-low-density lipoprotein cholesterol; WC, waist circumference.

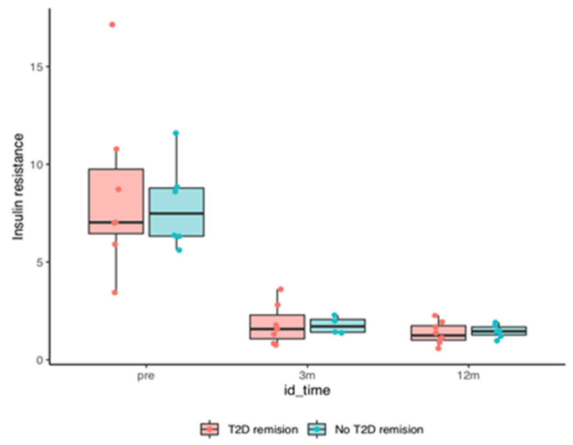

Figure S1. Behavior of the Homeostatic Model Assessment of Insulin Resistance (HOMA-IR) in obese patients before and 3 and 12 months after Roux en-Y gastric bypass according to the total type 2 diabetes remission. Legend. Boxplots comparing HOMA-IR between patients with (pink boxes; n= 8) and without (blue boxes; n = 6) remission of type 2 diabetes after Roux-en-Y gastric bypass, at preoperative (pre) and postoperative 3-months (3m) and 12-months (12m), where each color-matching dot corresponds to one individual. According to the Wilcox test, no changes were observed between these groups for all time points studied (preoperative, p = 0.950; postoperative 3-months, p = 0790; postoperative 12-months, p = 0.660).
